# Prognostic effect of preoperative serum estradiol level in postmenopausal breast cancer

**DOI:** 10.1186/1471-2407-13-503

**Published:** 2013-10-27

**Authors:** Ju-Yeon Kim, Wonshik Han, Hyeong-Gon Moon, Soo Kyung Ahn, Jisun Kim, Jun Woo Lee, Min Kyoon Kim, Taeryung Kim, Dong-Young Noh

**Affiliations:** 1 Department of Surgery, Gyeongsang National University Hospital, 90 Chilamdong, Jinju, Korea; 2 Department of Surgery and Cancer Research Institute, Seoul National University College of Medicine, 28 Yongon-dong, Seoul 110-744, Korea; 3 Department of Surgery, Gachon University Gil Hospital, 1198, Guwol-Dong, Incheon 405-760, Korea

**Keywords:** Estradiol, Postmenopause, Metastasis, Survival

## Abstract

**Background:**

The prognostic role of serum estrogen level in breast cancer patients is unclear. We investigated the prognostic importance of preoperative serum estradiol (E2) level in postmenopausal women according to their estrogen receptor (ER) status.

**Methods:**

The medical records of 313 postmenopausal breast cancer patients who underwent surgery between 2006 and 2008 at a single institution were retrospectively evaluated. Patients who received neoadjuvant chemotherapy, synchronous bilateral breast cancer, or those with metastasis at diagnosis were excluded. Serum E2 and follicular stimulating hormone (FSH) levels were measured by radioimmunoassay and immunoradiometric assay, respectively, within 3 months prior to surgery. After a median follow-up of 52.0 months (11–77 months), 21 women were found to have metastatic disease.

**Results:**

The overall, median E2 level was 13.0 pg/ml, and was slightly higher in ER-positive than ER-negative (p=0.69). The mean serum E2 level was significantly higher in patients with metastasis (17.41±8.34 pg/ml) than in those without metastasis (13.54±7.58 pg/ml) (p=0.02). Kaplan-Meier analysis using a cut-off of 13 pg/ml showed that, ER negative (p=0.02) but not ER positive (p>0.05) patients with higher E2 level showed significantly poorer metastasis-free survival. Multivariate analysis showed that, the high E2 level of ER negative tumors was an independent negative prognostic factor for metastasis- free survival (HR, 3.32; 95% CI, 1.05 to 10.51; p=0.04).

**Conclusions:**

Higher preoperative serum E2 level had a negative prognostic effect in postmenopausal women with breast cancer, especially in the ER-negative subgroup.

## Background

Many epidemiologic and experimental studies support an association between higher serum levels of sex steroid hormones and an increased risk of postmenopausal breast cancer, especially for estrogen receptor (ER)-positive breast cancers [1-6]. However, the prognostic role of the serum level of these hormones in newly diagnosed breast cancer patients is still unclear. Although several studies have reported that higher serum testosterone levels at diagnosis were associated with poor prognosis in postmenopausal women with breast cancer, other studies demonstrated no such association [7-9]. The role of testosterone in breast cancer has been attributed to its conversion to estrogen by aromatase.

Estrogen has been found to contribute significantly to breast tumor formation and growth [10-13]. High serum estradiol levels were reported to be associated with specific gene expression patterns in breast cancer tissue. [14]. In estrogen-dependent tumors, estrogen promotes cell proliferation and, suppresses apoptosis, by directly modulating gene transcription, making estrogen an important target in treatment [15].

ER status is important in breast cancer carcinogenesis and progression. Circulating estrogen binds to ER in breast cancer cells and stimulates cell division and growth. However, recent studies provided further molecular insights into the estradiol-dependent breast carcinogenesis, finding that estradiol may act independently of ER [16-18].

Serum estradiol level is significantly lower in postmenopausal than in premenopausal women. And postmenopausal women have been consistent in E2 levels without variation according to menstrual cycle. Here, we report the results of a single-institutional retrospective analysis of the prognostic importance of preoperative serum estradiol level in postmenopausal breast cancer patients. Our hypothesis is that serum estradiol level may be significant prognostic factor in postmenopausal breast cancer.

## Methods

**Figure 1:**
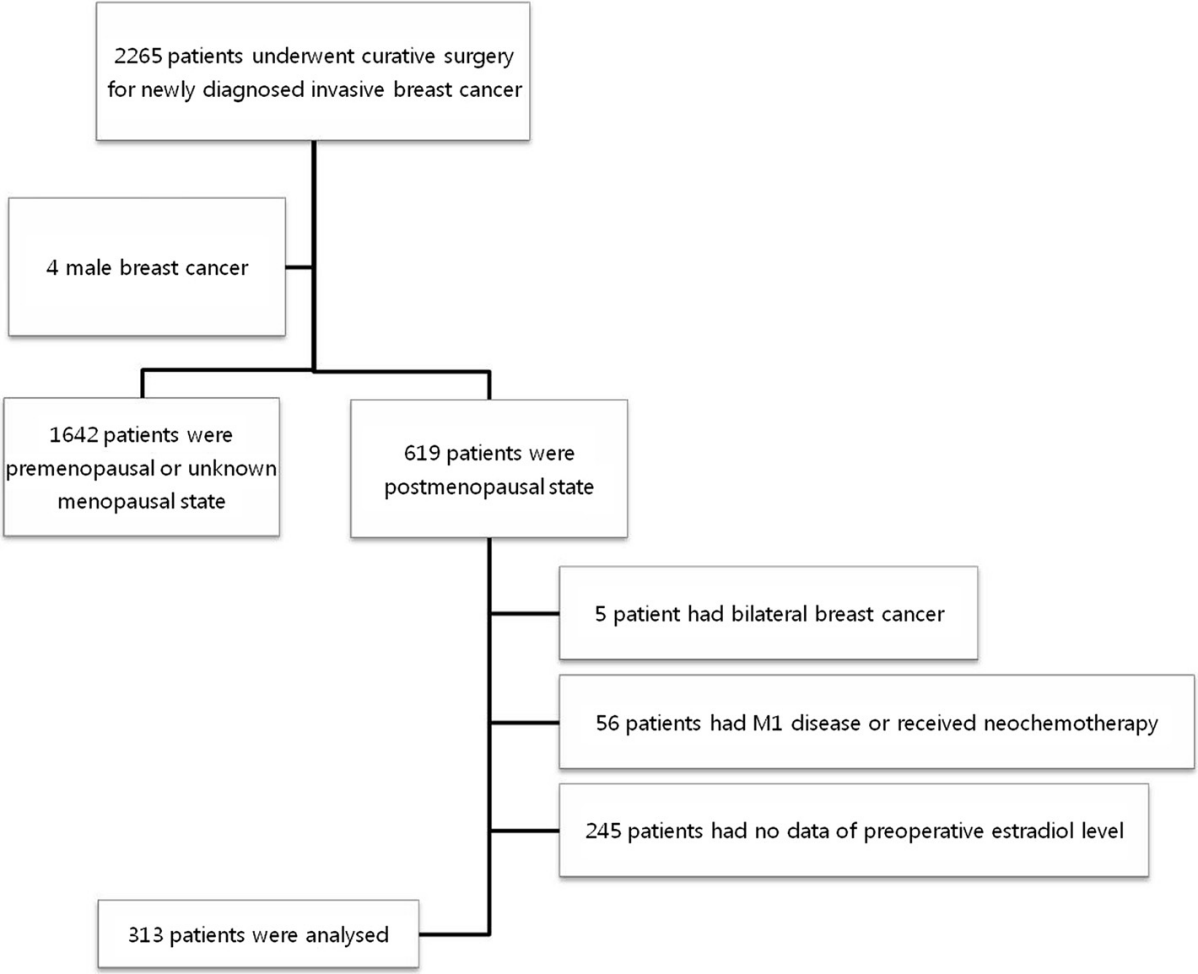
Flow chart of patients’ selection.

The Seoul national university hospital breast care center database was reviewed for the medical records of postmenopausal women who underwent curative surgery between September 2006 and December 2008 for newly diagnosed invasive breast cancer and for whom we had the information on serum estradiol levels within 3 months prior to surgery. Patients who received neoadjuvant chemotherapy, synchronous bilateral breast cancer or those with metastasis at diagnosis time were excluded from the study. Women were defined as postmenopausal if they had a bilateral oophorectomy, were aged 60 years or older, or were aged under 60 years with amenorrhea for at least 12 months, and their serum follicular stimulating hormone (FSH) levels satisfied the diagnostic criteria for menopause (FSH>30 mIU/mL). We identified 313 postmenopausal women eligible for the study (Figure 1).

Serum levels of E2 were measured by radioimmunoassay (RIA) using commercial kits (Biosource, Nivelles, Belgium), with intra- and inter-assay coefficients of variation (CV) of 4.9% and 5.2%, respectively. FSH were measured by immunoradiometric assay (IRMA) using commercial kits (Biosource), with a detection limit of 0.1 mlU/mL, and intra- and inter assay CVs of 3.3% and 7.1%, respectively.

Pathology data, including tumor size, grade, lymph node involvement, and immunohistochemistry results on hormone receptors expression, were reviewed. A cut-off value of 1% or more positively stained nuclei in the high-power fields was used to define ER and PR positivity. Patients were recommended to undergo adjuvant therapy and surveillance according to the St Gallen and/or NCCN guidelines.

Distant metastasis excluded local breast recurrence, axillary lymph node recurrence and newly diagnosed contralateral breast cancer. The time of metastasis was defined as the date confirmed by biopsy or image finding. Metastasis-free survival defined as the time period from the date of breast surgery to that of first diagnosis with distant metastasis by biopsy results or image or last follow-up. The Cox regression model was utilized to identify significant independent factors related to distant metastasis.

Student’s t-tests were used to compare E2 levels in two groups, and Pearson's correlation test was used to test the relationships between E2 level and age and body mass index (BMI). The Kaplan-Meier method and log-rank test were used for survival analysis. The Cox regression model was utilized to identify significant independent factors related to distant metastasis. The variables included in the final model were defined by backward selection. We excluded the missing or unknown data when we performed statistical analysis. Significance was defined as *p*<0.05. All statistical analyses were performed using SPSS (version 19.0 SPSS Inc., Chicago, IL, USA). Written informed consent was taken prior to surgery in all patients and the study protocol including the use of the database was approved by the Institutional Review Board of Seoul National University Hospital and met the guidelines of the responsible governmental agencies.

## Results

The clinical and histopathological characteristics of the studied patients are listed in Table 1. Patients ranged in age from 45 to 83 years, with 3 patients having undergone bilateral oophorectomy before diagnosis of breast cancer. Of the 195 patients with ER or PR positive tumors, 192 received adjuvant hormonal therapy.

**Table 1:** The clinical and histopathological characteristics of the included patients

**Characteristics**	**Number of patients (% of total)**			
	**Total**	**ER positive**	**ER negative**	**P value**
Enrolled patients	313	190	123	
Tumor size (mean), cm	2.17	2.03	2.40	
<2	170	121(63.7%)	49(39.8%)	<0.001
2-5	138	65(34.2%)	73(59.3%)	
≥5	5	4(2.1%)	1(0.8%)	
Nodal status				
0	206	130(68.4%)	76(61.8%)	0.310
1-3	81	44(23.2%)	37(30.1%)	
4-9	14	7(3.7%)	7(5.7%)	
≥10	12	9(4.7%)	3(2.4%)	
Nuclear grade				<0.001
Grade 1 or 2	127	109(57.4%)	18(14.6%)	
Grade 3	175	76(40.0%)	99(80.5%)	
Unknown	11	5(2.6%)	6(4.9%)	
Histologic grade				<0.001
Grade 1 or 2	131	113(59.5%)	18(14.6%)	
Grade 3	153	59(31.0%)	94(76.4%)	
Unknown	29	18(9.5%)	11(9.0%)	
PR status				<0.001
Positive	133	128(67.4%)	5(4.1%)	
Negative	180	62(32.6%)	118(95.9%)	
HER2/neu status				<0.001
Positive	45	9(4.7%)	36(29.3%)	
Negative	246	170(89.5%)	76(61.8%)	
Unknown	22	11(5.8%)	11(8.9%)	
Operation				0.014
Conservation	175	117(61.6%)	58(47.2%)	
Mastectomy	138	73(38.4%)	65(52.8%)	
Adjuvant chemotherapy				<0.001
No	131	103(54.2%)	28(22.8%)	
Yes	182	87(45.8%)	95(77.2%)	
Adjuvant Radiotherapy				0.129
No	133	74(38.9%)	59(48.0%)	
Yes	180	116(61.1%)	64(52.0%)	
Adjuvant hormonal therapy				
No		3(1.6%)		
SERM		36(18.9%)		
AI		137(72.1%)		
Switch*		14(7.4%)		
Distant metastasis		7(3.7%)	15(12.2%)	0.006

*Abbreviations*: *ER* estrogen receptor, *PR* Progesterone receptor, *SERM* selective estrogen modulator, *AI* aromatase inhibitor.

Switch*: Tamoxifen for 2–3 year, and then aromatase inhibitor to complete 5 year.

The overall median level of estradiol was 13.0 pg/ml, with mean estradiol levels higher in patients with ER positive than ER negative tumors (14.36±7.85 pg/ml *vs*. 12.97 ± 7.37 pg/ml, p=0.69) (Table 2). Serum levels of estradiol did not correlate with age (Pearson correlation coefficient=0.033, *p*=0.56) or BMI (Pearson correlation coefficient =0.106, *p*=0.06).

**Table 2:** Mean estradiol levels according to subgroups

		**Estradiol level (Mean±SD, pg/mL)**	**p value**
Tumor size	<2 cm	13.69±7.17	0.75
	≥2 cm	13.97±8.28	
Nodal status	N0	13.93±7.99	0.71
	N1-3	13.59±7.09	
ER	Negative	12.97±7.37	0.69
	Positive	14.36±7.85	
PR	Negative	13.41±7.52	0.27
	Positive	14.37±7.89	
Her-2	Negative	14.09±7.80	0.81
	Positive	14.40±7.33	
Histologic grade	Grade 1 or 2	13.91±8.05	0.80
	Grade 3	13.68±7.25	
Nuclear grade	Grade 1 or 2	13.78±8.38	0.89
	Grade 3	13.90±7.11	

*Abbreviations*: *SD* Standard deviation, *ER* estrogen receptor, *PR* Progesterone receptor.

During a median follow-up time of 52.0 months after diagnosis (range, 11 to 77 months), 3 patients had locoregioanl recurrence, 2 patients had contralateral breast cancer, and 21 patients had distant metastasis as the first event. And one patient with local recurrence eventually had distant metastasis. As the results, 22 patients were diagnosed with distant metastases, 7 in the ER positive and 15 in the ER negative group. Mean serum estradiol level was significantly higher in patients with than without metastasis (17.41±8.34 pg/ml *vs*. 13.54±7.58 pg/ml, *p*=0.02).

**Figure 2:**
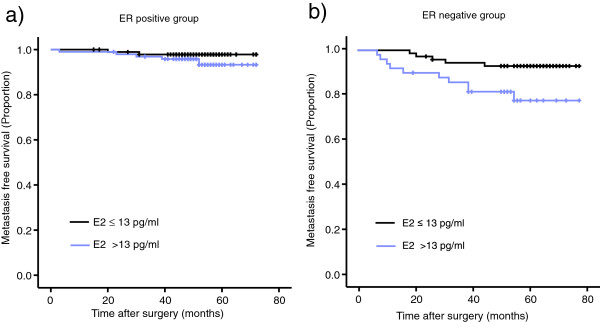
**Metastasis-free survivals according to ER status. a**. Metastasis-free survival was lower in ER-positive patients with higher than with lower estradiol level, although the difference was not statistically significant (Log-rank=0.25). **b**. In ER negative patients, higher estradiol levels were significantly associated with increased risk of metastasis (Log-rank=0.02).

We performed Kaplan-Meier survival analysis to evaluate the prognostic effect of estradiol according to ER status. Using the cut-off values 13 pg/ml, which is the median value in this study, we found that metastasis free survival was lower in ER-positive patients with higher than with lower estradiol level, although the difference was not statistically significant (Figure 2a). In ER negative patients, higher estradiol levels were significantly associated with increased risk of metastasis (Log-rank=0.02) (Figure 2b).

To confirm the prognostic effect of serum estradiol in ER negative breast cancer patients, we performed a multivariate analysis using a Cox hazard model. The estradiol level was found to be an independent negative prognostic indicator of metastasis free survival (HR, 3.32; 95% CI, 1.05 to 10.51; p=0.04) (Table 3).

**Table 3:** Univariate and multivariate analyses of prognostic factors including serum E2 level for metastasis-free survival according to ER status

	**ER positive group**						**ER negative group**					
	**Univariate analysis**			**Multivariate analysis**			**Univariate analysis**			**Multivariate analysis**		
	**HR**	**95% CI**	**p value**	**HR**	**95% CI**	**p value**	**HR**	**95% C**I	**p value**	**HR**	**95% CI**	**p value**
Age	1.06	0.97-1.16	0.24				1.02	0.95-1.09	0.63			
BMI, ≥23 kg/m^2^	1.22	0.52-2.86	0.64				1.02	0.37-2.82	0.97			
HER2/neu positive	0.99	0.98-1.01	0.69				0.97	0.33-2.84	0.96			
Tumor size, >2 cm	10.65	1.28-88.48	0.03	3.75	0.41-33.98	0.24	4.75	1.07-21.06	0.04	1.85	0.37-9.27	0.45
Node metastasis												
0	Ref.			Ref.			Ref.					
1-3	1.47	0.13-16.25	0.75	1.12	0.10-12.66	0.93	4.00	1.17-13.70	0.03	2.45	0.65-9.31	0.19
>3	18.96	3.47-103.63	0.001	9.05	1.50-54.45	0.02	10.70	2.67-42.90	0.001	2.99	0.62-14.36	0.17
Nuclear grade 3	3.06	1.06-8.83	0.04	1.21	0.08-19.62	0.87	1.11	0.53-2.34	0.78			
Histologic grade 3	3.59	1.24-10.38	0.02	8.04	0.54-120.81	0.13	1.16	0.55-2.45	0.69			
ELTE	2.91	0.65-13.00	0.16				8.28	2.63-26.06	<0.001	4.24	1.18-15.18	0.03
E2 level, >13 pg/ml	2.54	0.49-13.13	0.27				3.373	1.07-10.61	0.04	3.32	1.05-10.51	0.04
Chemotherapy	2.49	0.92-6.75	0.07									

A multivariate analysis was set by using all of the predictors with p values under 0.05 in univariate analysis.

*Abbreviations*: *ER* estrogen receptor, *BMI* Body mass index, *ELTE* endolymphatic tumor emboli, *E2* Estradiol.

We also found that, ER negative patients with higher estradiol levels were significantly associated with increased risk of diasease-sepecific events (including locoregional recurrence and contralateral recurrence). (Log rank=0.034) In multivariate analysis using a Cox hazard model, high E2 (>13 pg/ml) of ER negative tumors was negative prognostic factor for disease-free survival (HR, 2.717), but the p value was 0.058.

## Discussion

We have shown here that higher serum estrogen levels contribute to the risk of distant metastasis in postmenopausal breast cancer patients with ER negative tumors. Rock et al. [7] have also shown the significant association between serum estradiol level and patient’s survival in a nested case–control cohort of a randomized trial of diet intervention (Women’s Health Eating and Living study), although that study did not include subgroup analysis based on ER expression status.

Our observation, that serum estradiol level affects prognosis only in patients with ER negative tumors, was interesting since estradiol is thought to play a key role in the carcinogenesis of ER positive tumors. Indeed, our findings are supported by recent experimental studies showing that estradiol regulates the progression of ER negative breast cancer cell lines. For example, Gupta et al. [16] reported that estrogen promotes the growth, stromalization, and angiogenesis of an ER negative breast cancer cell line by systemic induction of host angiogenesis and bone marrow-derived stromal cell recruitment. Similarly, Banka et al. [17] showed that estradiol treatment of ovariectomized mice injected with an ER negative mouse mammary carcinoma cell line markedly increased the incidence of lung metastasis. These studies suggest that estradiol can act as a potent metastasis-promoter in ER negative tumors by a novel mechanism involving the host microenvironment. And another plausible explanation might be possibility that more aggressive ER negative breast tumors are associated with higher estrogen levels because they share a common cause, for example, such tumors could elicit a strong inflammatory response which both enables metastasis and also upregulates aromatase activity in surrounding tissues.

Indirect evidence supporting the role of estradiol in ER negative tumor development can also be found in human clinical studies. Ovariectomy was shown to signicantly reduce the incidence of both ER negative and ER positive tumors [19,20]. Prophylactic oophorectomy can prevent the development of breast cancers in BRCA1 mutation carriers in whom the main types of breast cancer are ER negative [21-23]. However, it is unclear whether it may be the consequence of inhibition of the transition of luminal of ER positive cells to negative or inhibition of tumorigenesis itself. And among BRCA1 mutation carriers older than age 50, no risk reduction was evident with prophylactic oophorectomy [23]. It is also unclear whether the estrogen lowering treatments like aromatase inhibitors were effective in ER negative tumors. According to the study of Jones et al. [24] which was central review of pathological specimens from patients entered in BIG 1–98 trials, aromatase inhibitors might have advantage in only patients whose tumor express ER. However, they didn’t measure the preoperative estradiol level, so it was impossible to compare the difference of effect according to estradiol level in patients with ER negative or positive tumors.

It is unclear why serum estradiol level was not significantly associated with the development of metastasis in ER positive breast cancer patients. One possible explanation is that almost all patients with ER positive tumors in our study cohort (98.4%) received anti-estrogen treatment with either tamoxifen or aromatase inhibitors. In addition, there were fewer events in ER positive tumors, so it might act as cause of limited power.

Premenopausal women experience changes in their serum estrogen levels throughout their menstrual cycles. In contrast, postmenopausal women have been more consistent in E2 levels, because of the absence of the variability of hormone levels with the menstrual cycle. Therefore, to analysis the difference of prognosis according to serum E2 level in premenopausal women, constant measure of E2 in time is important.

This study has several limitations. Due to its retrospective nature, there may have been a selection bias. Specially, the measurement of estradiol levels was not performed as a part of the prospective design. Some were measured for other studies, and others measured for standard element of the work-up for breast cancer by special physicians during different periods. Also, the median follow-up period of 52 months was not sufficient to assess the prognostic effect in breast cancer patients, especially in patients with ER positive tumors. And our findings are based on a quite small number of cases. Further confirmatory studies are needed.

## Conclusions

We found that a higher level of serum estradiol had a negative prognostic effect in postmenopausal women with ER negative breast cancer. These findings are hypothesis generating, suggesting that an estrogen-rich microenvironment can facilitate the progression of ER negative tumors. Additional in vivo and prospective cohort studies are needed to address this hypothesis.

## Abbreviations

ER: Estrogen receptor; E2: Estradiol; FSH: Follicular stimulating hormone; RIA: Radioimmunoassay; CV: Coefficients of variation; IRMA: Immunoradiometric assay; BMI: Body mass index.

## Competing interests

The authors declare that they have no competing interests.

## Authors’ contributions

All the authors have made substantial contributions to conception and design, acquisition of data, or analysis and interpretation of data. JYK conceived of and organized the study and was primarily responsible for drafting the manuscript. SKA and JK carried out collection of primary data and provided clinical input. JWL, MKK and TK confirmed patients’ outcomes of recurrence and follow up results and guided statistical analysus. HGM and NDY participated in the study design and helped to draft the manuscript. As responding author, WH designed and coordinated the research and provided close guidance throughout the process. All authors read and approved the final manuscript. The authors have been involved in drafting the manuscript or revising it critically for important intellectual content and have all given final approval of the version to be published.

## Pre-publication history

The pre-publication history for this paper can be accessed here:

http://www.biomedcentral.com/1471-2407/13/503/prepub
